# The Clinical Characteristics and Prognosis of Chinese Patients with Light-Chain Amyloidosis: A Retrospective Multicenter Analysis

**DOI:** 10.1007/s12288-021-01469-y

**Published:** 2021-07-28

**Authors:** Donghua He, Fangshu Guan, Minli Hu, Gaofeng Zheng, Jingsong He, Xiaoyan Han, Yang Yang, Pan Hong, Gang Wang, Yi Zhao, Wenjun Wu, Zhen Cai

**Affiliations:** 1 Department of Hematology, Bone Marrow Transplantation Center, The First Affiliated Hospital, School of Medicine, Zhejiang University, No. 79, Qingchun Rd., Hangzhou, 310003 Zhejiang Province China; 2 Department of Hematology, Taizhou Hospital of Zhejiang Province, Taizhou, China; 3 Department of Hematology, Shaoxing People’s Hospital, Shaoxing, China; 4 Department of Hematology, People’s Hospital of Quzhou, Quzhou, China

**Keywords:** Amyloidosis, Prognosis, Cardiac, Overall survival

## Abstract

To retrospectively identify the critical characteristics and prognostic factors of light-chain amyloidosis. Patients and Methods: Data were collected and compared from 91 patients who were diagnosed with light-chain amyloidosis at four hospitals between January 2010 and November 2018. We analyzed the clinical characteristics and performed an overall survival (OS) analysis. Results: Patients (median age, 60 years) were diagnosed with organ involvement of the kidney (91.2%), heart (56%), liver (14.3%), soft tissue (18.7%), or gastrointestinal tract (15.4%), and 68.1% of patients had more than two organs involved. Patients were most treated with bortezomib-based regimens (56%), and only one patient had autologous stem cell transplantation (auto-ASCT). The median OS was 36.33 months and was influenced by the ECOG score, renal involvement, cardiac involvement, hepatic involvement, and persistence of positive immunofixation. Patients who received bortezomib-based treatment had a trend of favorable OS compared to those who received non-bortezomib-based treatments, but the difference was not statistically significant. Although the overall number of organs involved was not related to OS, the number of organs involved in the heart, liver and kidney was related. Multivariate analysis indicated that cardiac involvement and negative hematologic response with persistence of positive immunofixation were independent prognostic factors for OS. Conclusion: Cardiac involvement and the hematologic response to treatment were independent prognostic factors for OS in light-chain amyloidosis patients.

## Introduction

Light chain (AL) amyloidosis is a disease derived from plasma cell clones, characterized by the extracellular deposition of N-terminally derived amorphous amyloid fibrils from a monoclonal light chain variable region. [1]. Despite having a relatively small size, a clone can set off devastating multiorgan damage, which is caused by the monoclonal light chain. The incidence of AL amyloidosis is estimated to be 1 case per 100,000 person-years in Western countries, there are approximately 1275–3200 new cases per year in the United States [2]. There is currently no official statistics on the incidence of AL amyloidosis in China. The symptoms are often varied and nonspecific, so the diagnosis of AL amyloidosis is often delayed. Many patients are diagnosed at an advanced stage, resulted in early mortality. The overall prognosis of AL amyloidosis is poor, mainly because amyloid deposits in important organs such as the heart, liver, and kidneys, and ultimately lead to organ failure and death. [3]. In the last 15 years, substantial progress has been made in understanding the biology of the amyloid plasma cell clones and the mechanisms of organ damage; yet there are no specific treatments can be used to treat amyloidosis. Mishra et al. found that human amyloidogenic light chain proteins directly cause cardiac dysfunction, so the circulating light chain can cause organ dysfunction [4]. Treatment approaches for AL amyloidosis focus on suppressing the abnormal plasma cell clone, with the aim of reducing the production of amyloidogenic light chains. Stem cell transplantation is still available for treatment, but this only applies to a small number of patients. For patients who are not suitable for transplantation, the standard treatment regimen is based on bortezomib treatment. For frail and older patients, oral treatment with melphalan and dexamethasone can be used. [5].

Many factors have been shown to be related to overall survival, such as the patient’s age, nutrition status, bone marrow plasma cell infiltration and the severity of organ involvement. The accumulation of clinical data from around the world may help improve the diagnosis, prognostic assessment, and treatment. At present, reports on the clinical characteristics and prognosis of AL amyloidosis in China are rare. Thus, we performed a retrospective analysis of 91 AL amyloidosis patients from four hospitals in China, try to find factors that affect the prognosis of AL amyloidosis. This study may enrich the existing database and contribute to future meta-analysis.

## Materials and Methods

We retrieved information on 91 patients who were diagnosed with primary systemic AL amyloidosis between January 2010 and November 2018 at the First Affiliated Hospital of Zhejiang University, Taizhou Hospital of Zhejiang Province, Shaoxing People’s Hospital, and People’s Hospital of Quzhou. Amyloidosis was diagnosed according to the updates of AL amyloidosis in 2020 [6], determined by the presence of Congo Red-positive fibril deposition and apple-green birefringence, as observed under polarized light. Immunohistochemistry was used to determine the kappa or lambda light chain specificity of the fiber. Immunofixation electrophoresis in serum or urine, serum-free light chain analysis or clonal plasma cell population in the bone marrow were required for the evaluation of clonality. Organ involvement was evaluated according to the consensus opinion from the 10th International Symposium on Amyloid and Amyloidosis [7]. Staging system for cardiac involvement was evaluated according to the 2004 version of the Mayo staging system [8]. The renal staging system was based on the combination of proteinuria and eGFR [9]. Patients with a positive family history of amyloidosis or amyloidosis secondary to multiple myeloma and lymphocytic proliferative diseases were excluded. This study was approved by the Ethics Committee of the First Affiliated Hospital of Zhejiang University, and was in accordance with the Declaration of Helsinki.

## Statistical Analysis

The overall survival (OS) was calculated from diagnosis until death or the last follow-up. Survival analysis for various factors were performed using the Kaplan–Meier method, and survival curves among groups was compared by the log-rank test. The risk of the selected variables was calculated using the Cox proportional hazards regression model, and 95% confidence intervals were generated, with a hazard ratio < 1.0 indicating survival benefit. Univariate and multivariate analyses were carried out to examine the predictive factors for OS. A 2-tailed P value of less than 0.05 was defined as significant. All statistical analyses were performed by SPSS 18.0.

## Results

### Symptoms and Clinical Presentations

**Fig. 1:**
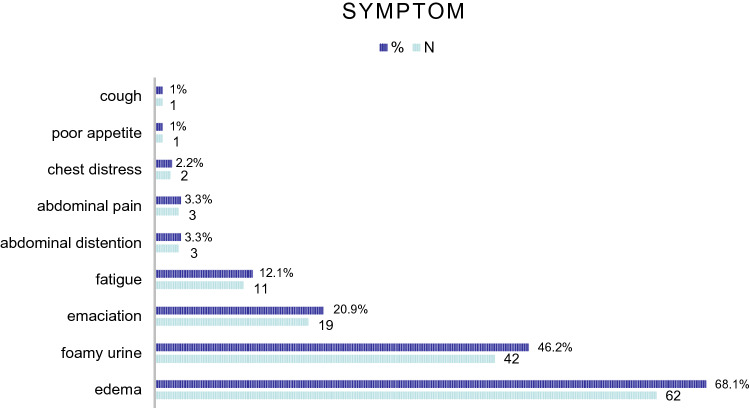
The incidence of symptoms at presentation

Ninety-seven patients were admitted to the three hospitals; 3 patients were lost to follow-up and 3 were excluded because the bone marrow plasma cells (BMPC) measurements at diagnosis were not available. A total of 91 patients were included in the analysis (Table 1). The median age at diagnosis was 60 years (41–82 years), and 56% of the patients were male. Edema and foamy urine were the two most common symptoms reported by patients at diagnosis, 62 (68.1%) and 42 (46.2%), respectively. 25 (27.5%) patients reported both symptoms. Nonspecific symptoms such as fatigue were present in 12.1% of the patients, and abdominal distention and abdominal pain were present in 6.6% of the patients. Only two patients presented with chest pain, one patient presented with poor appetite and one patient presented cough (Fig. 1). According to the Consensus Criteria [7], the kidney was involved in 83 patients (91.2%), the heart in 51 (56%), the liver in 13 (14.3%), the gastrointestinal tract in 14 (15.4%), the soft tissue in 17 (18.7%), the neurological system in 6 (6.6%) and the lung in 6 (6.6%). Among the patients, 29 (31.9%) had only one organ involved, 41 (45.1%) had two organs involved, and 21 (23%) had three or more organs involved.

**Table 1:** Baseline demographic, clinical characteristics and treatment

	Patients(n = 91)
Median age, year(range)	60 (41–82)
Male, n (%)	51 (56)
ECOG 2 or more, n (%)	32 (35.2)
Organ involvement, n(%)	
Renal	83(91.2)
Cardiac	51(56)
Hepatic	13(14.3)
Gastrointestinal	14(15.4)
Soft tissue	17(18.7)
Peripheral neuropathy	6(6.6)
Lung	6(6.6)
Number of organ involved	
1, n (%)	29 (31.9)
2, n (%)	41 (45.1)
≥ 3, n(%)	21 (23)
Heavy chain	
IgG, n (%)	21 (23.1)
IgA, n (%)	20 (22.0)
Light-chain isotype	
Kappa, n (%)	31 (34.1)
Lambda, n (%)	60 (65.9)
Immunofixation	
Serum + Urine + , n (%)	55(60.4%)
Serum + Urine-, n (%)	7(7.7%)
Serum - Urine + , n (%)	20(22%)
Serum - Urine-, n (%)	9(9.9%)
Mayo Stage 2004, n (%)	
Stage I/II/III	19(20.9)/22(24.2)/14(15.4)
Missing	36(39.6)
Renal Stage, n (%)	
Stage I/II/III	33(36.3)/37(40.7)/9(9.9)
Missing	12(13.2)
Proteinuria	
< 0.5 g/24 h, n (%)	6(6.6%)
0.5–5 g/24 h, n (%)	42(46.2%)
> 5 g/24 h, n (%)	31(34.1%)
Missing, n (%)	12(13.2%)
eGFR	
≥ 50(ml/min), n (%)	73(80.2%)
< 50(ml/min), n (%)	18(19.8%)
BMPCs, median (range)	5(0–14)
Hb(g/L), median (range)	125 (79–190)
NTproBNP(pg/mL), median (range)	1195 (14–9000)
TnI(ng/ml), median(range)	0.025(0–8.4)
Serum albumin(g/L), median (range)	26.8 (10.5–47.4)
Creatinine(μmol/L), median (range)	79(29–579)
Treatment	
VD/VCD, n (%)	51(56.0)
TCD, n (%)	24(26.4)
MP, n (%)	2(2.2)
RD, n (%)	3(3.3)
Other (including no-treatment), n (%)	11(12.1)

Abbreviations: BMPCs = bone marrow plasma cells;

VD/VCD = Bortezomib + dexamethasone/Bortezomib + cyclophosphamide + dexamethasone; TCD = thalidomide + cyclophosphamide + dexamethasone; MP = Melphalan + prednisone; RD = Lenalidamine + dexamethasone

### Biopsy and Laboratory Findings

**Fig. 2:**
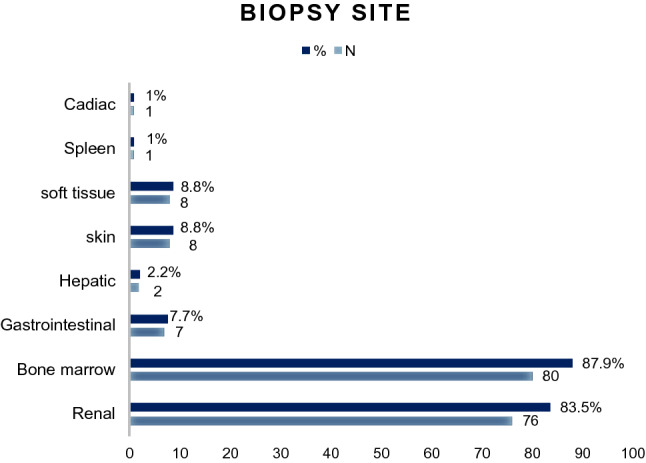
The biopsy site(s) of all patients

During the diagnostic workup, 80 patients (87.9%) underwent bone marrow (BM) biopsy, and 76 patients (83.5%) underwent kidney biopsy. However, only 8 (8.8%) patients underwent skin biopsies, 4 patients (4.4%) underwent a soft tissue biopsy. Liver and gastrointestinal tract biopsies were performed in 2 (2.2%) and 7 (7.7%) patients, respectively (Fig. 2). All the patients had immunohistochemical staining of amyloid deposits for the κ or λ light chains: 60 (65.9%) of the patients stained positively for λ and 31 (34.1%) for κ light chains. Overall, 82 patients (90.1%) had a monoclonal protein detected by serum or urine immunofixation. Among them, 61 patients had monoclonal proteins on serum immunofixation and 75 patients had monoclonal proteins on urine. In detail, 55 patients (60.4%) had positive immunofixation in both serum and urine, 7 patients (7.7%) were serum positive but urine negative, 20 patients (22%) were urine positive but serum negative, and 9 patients (9.9%) were both serum and urine negative. The immunoglobulin heavy chain was IgG in 21 (23.1%) patients and IgA in 20 (22%) patients; a single light chain was found in 50 (54.9%) patients. The median bone marrow plasma cell infiltration was 5%. Anemia was rare, with a median hemoglobin level of 125 g/L. Most patients (67%) had hypoproteinemia with serum albumin less than 30 g/L, and the median was 26.8 g/L. The median level of creatinine was 79 μmol/L. The total 24-h urine protein of 48 (52.7%) patients was less than 5 g, and 31 (34.1%) patients were greater than 5 g. 18 (19.8%) patients had eGFR greater than/equal to 50 ml/hour, 73 (80.2%) patients had eGFR less than 50 ml/hour. In the cardiac assessment, the median level of the N-terminal pro-brain natriuretic peptide (NT-proBNP) was 1195 pg/ml, the median level of TropI was 0.025 ng/ml.

### Treatment and Survival

Patients were treated with several different regimens (Table 1). Bortezomib- based regimens (VD/VCD = bortezomib + dexamethasone/bortezomib + cyclophosphamide + dexamethasone) were used as the primary treatment in most patients (56%); thalidomide-based regimens were used in 26.4% of patients, and 3.3% of patients received lenalidomide-based regimens. A melphalan-based regimen was used in 2 (2.2%) patients. 10 patients (11%) did not receive any treatment, and only1 patient (1.1%) received HDM-ASCT treatment. The median OS from diagnosis was 36.33 months (range, 0.7–113.17 months), the median follow-up period was 31.9 months.

### Univariate and Multivariate Analysis of OS

**Fig. 3:**
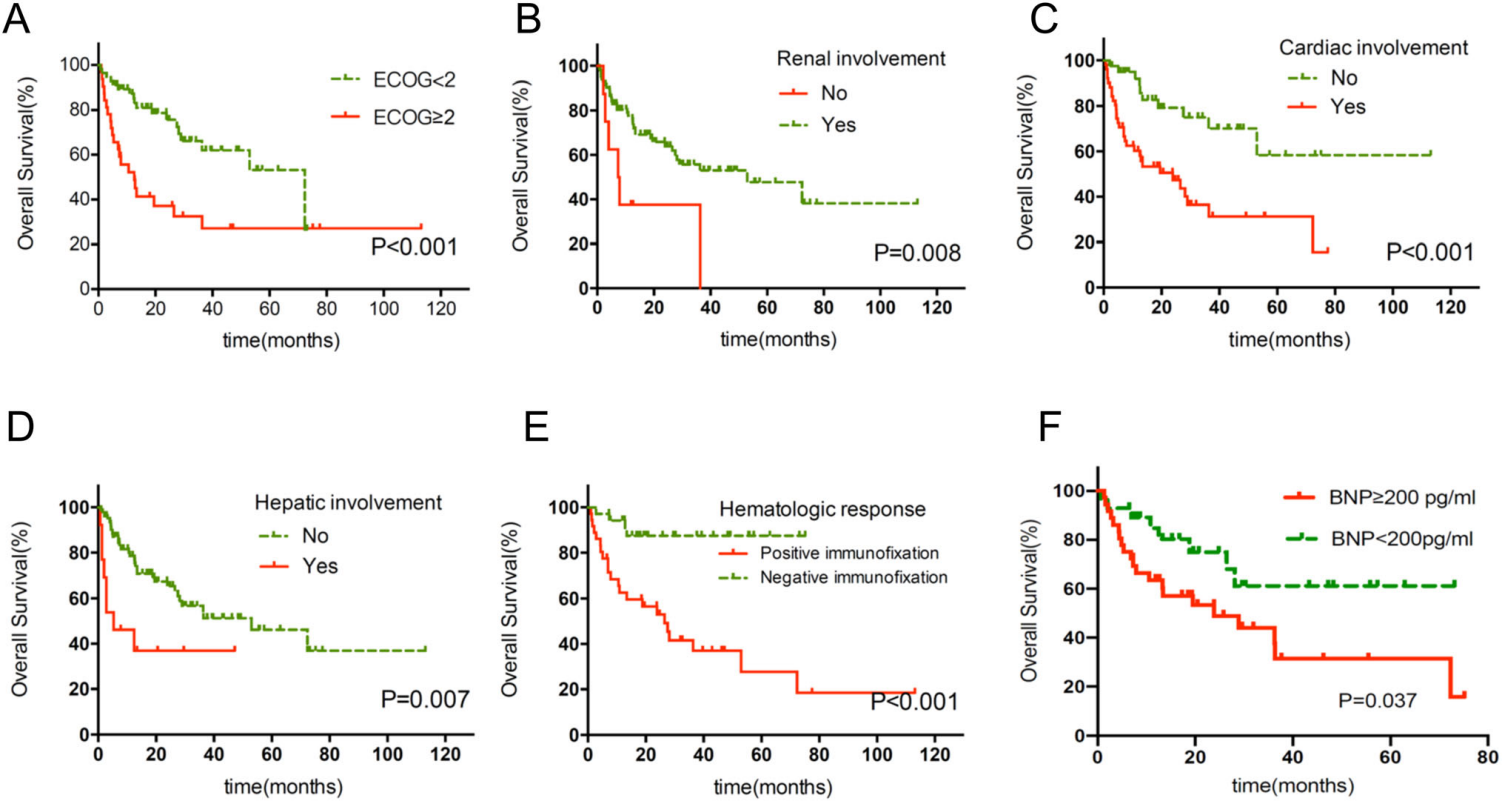
Kaplan–Meier curves demonstrating differences in overall survival. **a** Survival of patients according to ECOG: ECOG ≥ 2 versus ECOG < 2 (*P* = 0.002). **b** Survival of patients with and without renal involvement (*P* < 0.001). **c** Survival of patients with and without hepatic involvement (*P* = 0.002). **d** Survival of patients with and without cardiac involvement (*P* = 0.032). **e** Survival of patients according to immunofixation results (*P* < 0.001). **f** Survival of patients according to BNP: BNP ≥ 200 pg/ml vs BNP < 200 pg/ml (*P* = 0.037)

Table 2 summarizes the correlation between patient clinical characteristics and prognosis (OS). A univariate log-rank test showed that certain patient characteristics had a significant effect on OS (*P* value range < 0.001–0.008). An ECOG score ≥ 2 (12.53 months vs 72.33 months; *P* = 0.001, Fig. 3a), renal involvement (52.97 months vs 7.3 months; *P* = 0.008, Fig. 3b), cardiac involvement (23.9 months vs not reached; *P* = 0.001, Fig. 3c), hepatic involvement (52.97 months vs 5.3 months; *P* = 0.007, Fig. 3d), negative hematologic response with persistence of positive immunofixation (26.46 months vs not reached; *P* < 0.001, Fig. 3e), and BNP ≥ 200 pg/ml (23.9 months vs not reached; *P* = 0.037, Fig. 3f) were associated with inferior survival.

**Table 2:** Effect of patient characteristics on overall survival based on univariate analysis

Characteristic	HR	95% CI	*p* value
Age > 65 years	1.719	0.867–3.409	0.117
Sex (male)	1.171	0.624–2.197	0.623
ECOG ≥ 2	2.802	1.485–5.289	0.001
Renal involvement	0.320	0.132–0.774	0.008
Cardiac involvement	3.331	1.621–6.845	0.001
Hepatic involvement	2.828	1.290–6.201	0.007
3 or more organ involved	1.943	0.830–4.544	0.119
eGFR(< 50mi/min/1.73m^2^)	1.840	0.911–3.716	0.084
Serum albumin(< 30 g/L)	0.746	0.396–1.408	0.365
Bortizomib treatment	0.560	0.276–1.136	0.108
Light-chain isotype (kappa)	1.179	0.615–2.261	0.62
Mayo Stage 2004(I vs II/III)	0.499	0.215–1.161	0.107
NT-proBNP(≥ 1800 pg/ml)	1.855	0.895–3.846	0.092
BNP(≥ 200 pg/ml)	2.32	1.026–5.247	0.037
TnI(≥ 0.08 ng/ml)	1.088	0.514–2.305	0.825
Hematologic response: Positive immunofixation	6.464	2.224–18.785	< 0.001

**Fig. 4:**
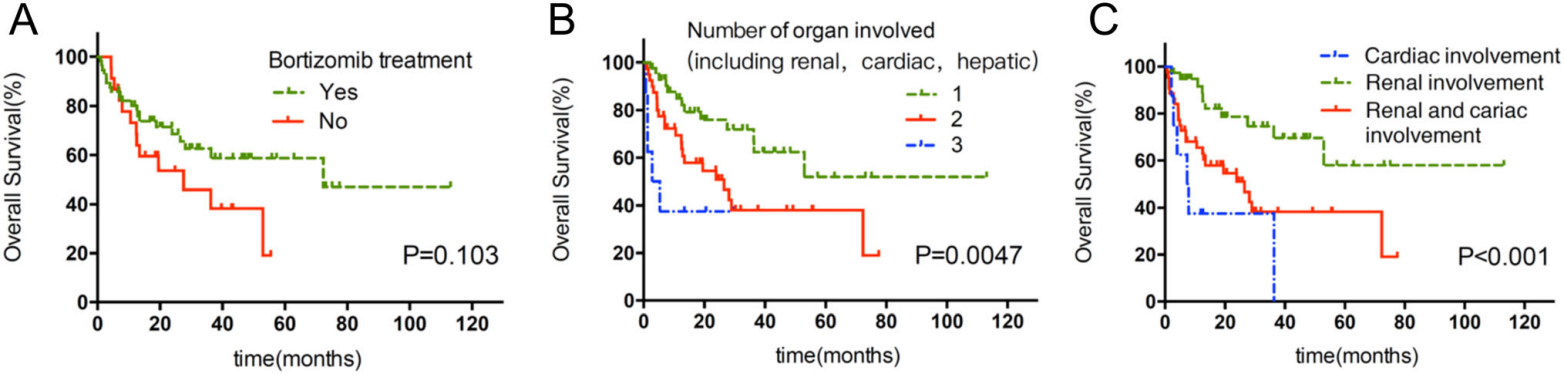
Overall survival of patients according to the treatment group and organ involvement. **a** With and without bortezomib treatment (*P* = 0.103). **b** Numbers of organ involved, including cardiac, renal and hepatic involvement (1 vs 2 *P* = 0.017; 1 vs 3 *P* = 0.002; 2 vs 3 *P* = 0.172). **c** Renal involvement, cardiac involvement, concurrent renal and cardiac involvement (renal vs cardiac *P* = 0.000; renal vs concurrent *P* = 0.003; cardiac vs concurrent *P* = 0.167)

Patients who had been treated had a significantly longer OS than that of patients who did not receive any treatment (52.97 months vs 4.9 months; *P* = 0.002). Patients who received bortezomib-based treatment had a trend of favorable OS compared to those who received non-bortezomib-based treatments, although the difference was not statistically significant (72.33 months vs. 39.411 months; *P* = 0.103, Fig. 4a). Although the overall number of organs involved was not related to OS, the number of organs involved in the heart, liver and kidney was related. The involvement of one of these organs was associated with better OS than that in patients in whom two or three of these organs were involved (not reached vs 26.46 months vs 2.77 months; *P* = 0.0047; Fig. 4b). Patients with cardiac involved alone have shorter OS than those patients with renal involved alone. Patients with both cardiac and renal involvement had a better OS than the group with cardiac involved alone but had a worse OS than that in the group with renal involved alone (26.46 months vs 7.3 months VS not reached; *P* < 0.001, Fig. 4c).

**Fig. 5:**
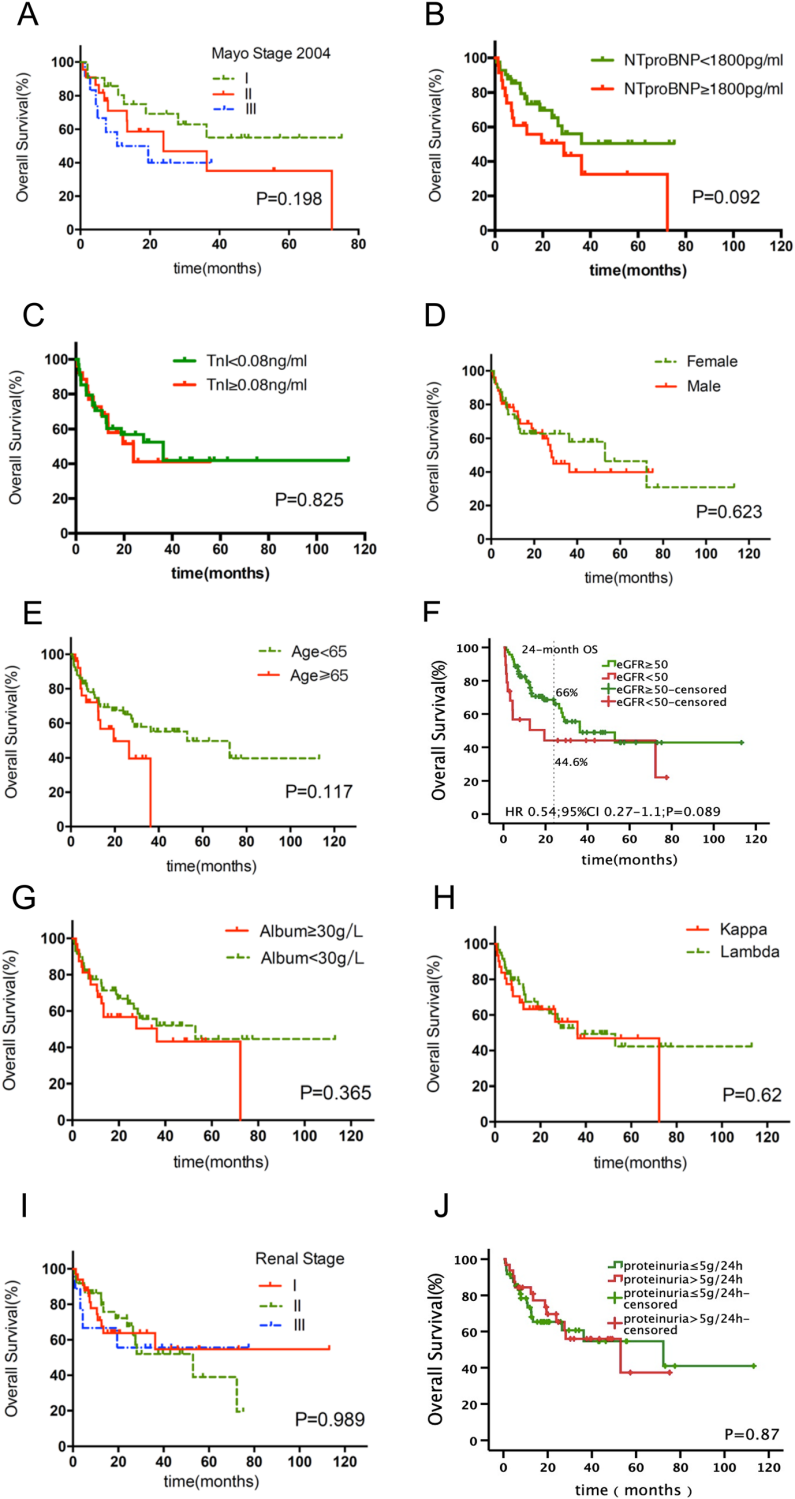
Overall survival of patients according to **a** the Mayo stage 2004 (stage I vs stage II *P* = 0.193; stage I vs stage III *P* = 0.116; stage II vs stage III *P* = 0.490), **b** NT-proBNP (*P* = 0.092), **c** TnI (*P* = 0.825), **d** sex (*P* = 0.623), **e** age (*P* = 0.117), **f** eGFR (*P* = 0.084), **g** albumin (*P* = 0.365), **h** light-chain isotype (*P* = 0.62) and **i** renal stage (*P* = 0.989), **j** proteinuria (*P* = 0.87)

Because the detection technology was limited in a few hospitals until 2017, only a small number of patients have detected the serum free light chain. Hence, we used the 2004 version of the Mayo staging system rather than the 2012 one. The results showed that the 2004 staging (not reached vs 23.9 months vs 10.57 months, *P* = 0.198, Fig. 5a), NT-proBNP (not reached vs 28.93 months, *P* = 0.092, Fig. 5b) and TropI levels (36.3 months vs 23.9 months, *P* = 0.825, Fig. 5c) were not related to OS. Furthermore, the patient gender, age, eGFR, serum albumin level, light-chain type, kidney stage, TropI level and proteinuria were not predictors of OS (Fig. 5d–j).

The multivariate analyses indicated that cardiac involvement and negative hematologic response with persistence of positive immunofixation were independent prognostic factors for OS (*P* < 0.05) (Table 3).

**Table 3:** Effect of patient characteristics on overall survival based on multivariate analysis

Characteristic	HR	95% CI	*p* value
ECOG ≥ 2	1.376	0.56–3.378	0.487
Renal involvement	0.41	0.146–1.148	0.09
Cardiac involvement	3.15	1.253–7.916	0.015
Hepatic involvement	3.026	0.83–11.033	0.093
Hematologic response: Positive immunofixation	3.15	1.253–7.916	< 0.001

## Discussion

Immunoglobulin light-chain amyloidosis is the most common form of amyloidosis, accounting for approximately 70% of all cases of these diseases [10]. The Medicare claims database showed that the median age at diagnosis for light-chain amyloidosis is 63 years, the incidence of 10–14 patients per million per year, and the prevalence higher in males than in females. The median age of the 91 patients in this study was 60-years-old, which is a little younger than that in Western countries. Because this disease can affect many organs and patients’ symptoms are usually nonspecific, early and accurate diagnosis is usually not possible. An online survey of the Amyloid Research Consortium found that 37% of patients were diagnosed one year after the onset of initial symptoms and that an average of three doctors were seen before diagnosis. Among the patients we examined, edema and foamy urine were the two most common symptoms reported, and it is difficult for us to recognize this disease based on these symptoms. In this study, 68.1% (62 out of 91) of patients had more than two organs involved at diagnosis, which is similar to the rate of 65% (54 of 84) that was reported in South Korea [4], and higher than the rate of 60.3% (38 of 63) reported in Denmark [11]. This result may be because some primary hospitals in China, physicians feel difficult to recognize the disease and its symptoms, thus delayed diagnosis. Therefore, efforts should be made to educate primary care physicians to have better awareness of the disease.

In our study, kidney involvement in almost all patients was confirmed by biopsy. In fact, an abdominal fat pad aspirate is a simple and minimally invasive procedure. In combination with the bone marrow biopsy, which was stained with Congo red, and/or the biopsy of a minor salivary gland, this procedure can yield a diagnostic sensitivity of approximately 90%; thus, removing the need for organ biopsies [12–14]. Skin and soft tissue biopsies were only conducted in 18.7% (16/91) of the patients in these patients. We might try to avoid many invasive procedures in the future.

The patients who were diagnosed between 2004 and 2015 at the Pavia Amyloidosis Research and Treatment Center demonstrated that the heart is the most affected organ, followed by the kidneys. We found that the kidney involvement rate in our analysis was 91.2%, which was higher than that in other published studies (71–57%) [15]. Another Chinese center even reported that 100% of patients had renal involvement [16]. These differences may be because in China, those patients who do not have renal involvement have a higher missed diagnosis rate compared to patients who have renal involvement (for example, those who do not have renal injury are unlikely to undergo cardiac biopsy, cardiac MRI, or liver puncture, and therefore, are more likely to have a missed diagnosis). Some studies have shown that renal insufficiency leads to unfavorable outcomes [17], but other studies showed that renal AL had a better 5-year survival rate, ranging from 60–80% [9]. In our study, patients in the eGFR-reduced group had higher early mortality, and the 2-year OS of the two groups was 66% versus 44.6%, respectively. However, due to the small number of patients with eGFR less than 50, there was no statistical difference in long-term OS between the two groups. In general, our results did not show a significant correlation between renal involvement and OS, and other kidney-related indicators (such as eGFR, proteinuria, album, renal stage) also do not show a significant correlation with OS.

The presence and severity of heart involvement is the most important prognostic determinant, and the main cause of early death [18]. The rate of cardiac involvement in our study was like that in previous reports [17]. A data from European reported that heart-involved patients had a survival rate less than 30% beyond 3 years, with a median survival of 7.1 months. Our study showed that the 3-year OS rate for patients with cardiac involvement was 36.4%, with a median survival of 23.9 months, which was like that in a previous Chinese study that reported a 40% 3-year OS and an 18-month median survival [16]. A recent meta-analysis demonstrated that bortezomib treatment significantly improved the overall response rate (ORR), complete response, cardiac response rate, 2-year overall survival and the risk of neuropathy and reduced overall mortality compared to those in the controls without bortezomib therapy [19]. Our results did not show a difference between the two groups, probably because the number of people in the two groups differed greatly.

Hepatic involvement is another poor prognostic factor, with a median OS of only 5.3 months in this study, which is much worse than that in the cardiac involvement group. Recently, a study from Italy compared the clinical presentation and outcome of 225 patients with and 643 patients without liver involvement from a series of 868 consecutive patients with light-chain amyloidosis who were diagnosed between 1986 and 2007. This study demonstrated that liver involvement was the hallmark of aggressive disease in light-chain amyloidosis [20]. But in our study, whether the liver is involved is not the main factor affecting OS.

Badar et al. [21] found that the overall survival of the patients with both cardiac and renal involvement was comparable to that of patients with cardiac involved alone but was worse than that of patients with renal involved alone. We found that the OS of the both cardiac and renal group was better than that of the cardiac alone group but was worse than that of the renal alone group. We speculated that this result was observed due to the popularity of kidney biopsy, so that the cardiac involvement of patients in both the cardiac and kidney involvement group was discovered early, and its severity was lower than that in the cardiac alone group.

Previous reports have claimed that the hematologic response is very important for evaluating the treatment efficacy and predicting survival. Because only a small number of patients have detected the serum free light chain, we were unable to accurately assess the hematological response. We found that the patients with a persistence of positive immunofixation after treatment had a shorter OS than that of patients with negative immunofixation (26.46 months vs not reached). We believe that as the technique improves and becomes more readily available, we will perform the serum free light chain test in a timely manner in prospective clinical work, and our future assessments will be more accurate. Until then, this result may provide a viable method for predicting prognosis in areas or hospitals that do not have conditions to detect serum free light chains.

It was reported that the Mayo stage and the level of NT-proBNP/BNP and troponin levels were related to the prognosis of light chain patients [22, 23]. Feng J et al. found that although univariate analysis shows TropI and NT-proBNP are risk factors for poor prognosis, TropI in multivariate analysis is not [22]. Ishiguro K et al. examined the effect of BNP on survival with a cut-off value of 1000 pg/mL at diagnosis, but could not detect significant survival difference in both groups. Their conclusion is the decline of BNP to 200 pg/mL or less during treatment was a predictor of survival in patients with cardiac AL amyloidosis [23]. Our study showed that although the Mayo stage 2004, NT-proBNP and TropI levels do not correlate patient survival, BNP ≥ 200 pg/ml at diagnosis was associated with inferior survival. This may because serum NT-proBNP levels are more affected than BNP by a renal function that is often impaired in AL patients. Other studies have found that the number of organs involved (more organs were worse) was a poor prognostic factor. In our data, the overall number of organs involved was not related to OS, but the number of organs that are involved among the heart, kidney and liver is correlated with the prognosis.

To date, only a few studies have focused on light-chain amyloidosis in China. The small number of studies could be because the incidence of light-chain amyloidosis is low, and only some large hospitals can make the diagnosis. The patients’ status and treatment also vary across centers. Some patients did not receive therapy when the diagnosis was established, mainly due to financial concerns or the severity of the organ involvement. In this study, between 2010 and 2018, approximately 10% of patients did not receive any treatment. Those patients had a worse clinical outcome, with a median OS of 5.1 months, which was less than the OS that was reported previously, compared to that of patients who did receive treatment [24]. In our study, 56% of patients received bortezomib-based regimens, such as VCD or VD, but only one patient received ASCT, which was mainly attributed to the patient status and cardiac involvement. ASCT can induce durable hematological and organ responses in systemic light-chain amyloidosis [25]. However, because of the small number of patients who received ASCT in our study, we could not analyze this factor. In addition, we also found that albumin and light-chain type did not affect patient survival in this study. We did only few FISH tests for these patients, so this factor could not be used as a prognostic factor in our data.

Because many patients did not receive regular treatment, or returned to the local hospital for treatment, or did not continue to come to the hospital for follow-up after the disease improved, it was impossible to obtain patient PFS and response evaluation (both hematological and organ response) related data. In future clinical work, we need to strengthen the management and follow-up of patients and obtain more abundant clinical data to guide clinical work.

## Conclusion

This retrospective study reports the detailed clinical presentations of AL amyloidosis patients and prognostic factors at four hospitals in China. Cardiac involvement and negative hematologic response with persistence of positive immunofixation were independent prognostic factors for OS in AL amyloidosis patients. The number of organs that are involved among the heart, kidney and liver is correlated with the prognosis. Although the lack of serum free light chain and the small sample size are important defects of the study, these results may help to improve the treatment and prognosis of AL amyloidosis, as well as to provide data that will aid future meta-analysis.
